# Supplementary material for: Child Welfare Investigations of Exposure to Intimate Partner Violence Referred by Medical Professionals in Ontario: A Uniquely Vulnerable Population?
Source: Healthcare (Basel). 2023 Sep 21;11(18):2599. doi: 10.3390/healthcare11182599 (PMC10530827; doi:10.3390/healthcare11182599)
Supplement: Supplementary file 1 [file healthcare-11-02599-s001.zip › healthcare-2562543-supplementary.pdf]

Table S1. List of variables used in bivariate analyses

Variable	Definition
Referral source	Refers to the investigation's referral source (e.g., medical, police, school, parent, etc.)
Medical referral source	Refers to investigations where the source of the referral was a hospital worker, community health nurse, or community physician.
Child age	Refers to the age of each child living in the home at the time of the investigation.
Child ethnicity/race/Indigeneity	Refers to ethnicity/race/Indigeneity of the investigated child
Child functioning concerns	
Developmental/physical	Refers to investigations where the worker identified the presence of a suspected or confirmed developmental or physical functioning concern for the child (i.e., positive toxicology at birth, Fetal Alcohol Spectrum Disorders, failure to meet developmental milestones, intellectual or developmental disability, physical disability, or academic/learning disability).
Emotional	Refers to investigations where the worker identified the presence of a suspected or confirmed emotional functioning concern for the child (i.e., attachment issues, depression/anxiety/withdrawal, suicidal thoughts, suicide attempts, or self-harming behaviours).
Behavioural	Refers to investigations where the worker identified the presence of a suspected or confirmed behavioural functioning concern for the child (i.e., ADHD, aggression, inappropriate sexual behaviour, alcohol abuse, drug abuse, running away from home, Youth Criminal Justice Act Involvement)
Primary caregiver age	Refers to the age of the primary caregiver during the time of the investigation.
Primary caregiver cooperative	Refers to investigations where the worker considered the primary caregiver to be cooperative, as opposed to non-cooperative or not contacted.
Primary caregiver risk factors	Refers to investigations where the investigating worker noted the presence of a suspected or confirmed pimary caregiver risk factor(s) (e.g., alcohol abuse, drug abuse, mental health issues, few social supports).
Household income source	Refers to the primary source of household income such as full-time, part-time, or seasonal employment, and employment insurance or other benefits.
Number of moves	Refers to the number of times in the past year the household moved
Overcrowding	Includes investigations where, in the investigating worker's clinical opinion, the household was overcrowded.
Ran out of money for basic necessities	Includes investigations where in the last six months, the household ran out of money for basic necessities (i.e., housing, utilities, phone, transportation, medical care)
Emotional harm	Refers to investigations where there was evidence that the investigated child was mentally or emotionally harmed as a result of substantiated maltreatment.
Physical harm	Refers to investigations where there was evidence that a child was physically harmed.
Previous investigations	Includes investigations where a family has been previously investigated by a child welfare agency.
Substantiation	Refers to investigations where, in the worker's clinical judgement, the child was a victim of child maltreatment.
Transfer to ongoing services	Refers to investigations kept open to provide ongoing child welfare services following the initial investigation.
Referrals to non-child welfare services	Refers to investigations where a worker made a referral to a non-child welfare service either internal or external to their organization (e.g., food bank, mental health services).

Placement	Refers to cases where a child was placed in out-of-home care during the investigation.
